# Recent advances in understanding and managing dystonia

**DOI:** 10.12688/f1000research.13823.1

**Published:** 2018-07-24

**Authors:** Stephen Tisch

**Affiliations:** 1 Faculty of Medicine, University of New South Wales, Sydney, Australia; 2 Department of Neurology, St Vincent’s Hospital, Sydney, Australia

**Keywords:** dystonia, cerebellum, deep-brain stimulation, stereotactic radiosurgery, functional magnetic resonance imaging

## Abstract

Within the field of movement disorders, the conceptual understanding of dystonia has continued to evolve. Clinical advances have included improvements in recognition of certain features of dystonia, such as tremor, and understanding of phenotypic spectrums in the genetic dystonias and dystonia terminology and classification. Progress has also been made in the understanding of underlying biological processes which characterize dystonia from discoveries using approaches such as neurophysiology, functional imaging, genetics, and animal models. Important advances include the role of the cerebellum in dystonia, the concept of dystonia as an aberrant brain network disorder, additional evidence supporting the concept of dystonia endophenotypes, and new insights into psychogenic dystonia. These discoveries have begun to shape treatment approaches as, in parallel, important new treatment modalities, including magnetic resonance imaging-guided focused ultrasound, have emerged and existing interventions such as deep brain stimulation have been further refined. In this review, these topics are explored and discussed.

## Tremor in dystonia

A relatively recent development in dystonia has been increasing interest in and recognition of dystonic tremor. In support of this observation, the number of published articles concerning dystonic tremor has steadily increased. The most recent revised dystonia classification ^1^ specifically includes tremor as a potential feature of dystonia. However, defining tremor in dystonia has proven both difficult and controversial. The most recent tremor classification ^2^ uses dual axes of clinical features and etiology, similar to the revised dystonia classification. In the current classification, dystonic tremor can be diagnosed only when tremor is present in a body part also affected by dystonia. Tremor present in a body region unaffected by dystonia is defined as tremor associated with dystonia (TAWD). The current classification no longer retains the category “dystonia-gene associated tremor” found in the previous tremor classification ^3^. It is worth noting that the current tremor classification, particularly as it relates to tremor in dystonia, remains somewhat controversial and not universally accepted. Some of the more recently discovered monogenic isolated dystonias, including *ANO3* ^4^ and *GNAL* ^5^, frequently manifest tremor. Dystonic tremor is clinically distinguishable by asymmetry, jerkiness, task and position specificity, and the frequent presence of null points where tremor diminishes or even disappears in certain positions of the affected body part. Recent large studies of adult idiopathic isolated focal and segmental dystonia have shown that tremor is common, most frequently involves the head followed by the upper limbs, and appears to be associated with the spread of dystonia to other body regions ^6– 8^. Rest tremor, in dystonia, is not uncommon and usually involves the arm ^9^, creating potential diagnostic confusion with Parkinson’s disease ^10^. Under the current tremor classification, patients with pure tremor of the dystonic type are not considered to have dystonic tremor or TAWD, and an alternative designation, “indeterminate tremor”, has been proposed ^11^. Increasingly, movement disorder specialists recognize and diagnose pure dystonic tremor and distinguish it from essential tremor, although the clinical distinction may be difficult. Historically, there has been a tendency to over-diagnose essential tremor among patients with dystonic tremor, resulting in misclassification ^12, 13^. The apparent coexistence of “essential tremor” and focal dystonia has long been recognized ^14^ and continues to be described ^15^, but it is more likely that these patients have dystonic tremor or TAWD rather than dual diagnosis of essential tremor.

Differentiation of dystonic tremor from essential tremor is clinically important because dystonic tremor behaves differently in its natural history and response to therapy and may have additional genetic implications. Pathophysiological differences between dystonic tremor and essential tremor may explain its differing clinical behavior. Electrophysiological tremor studies identify dystonic tremor as being more irregular and having varying duration and intensity of bursts of muscular activity ^16^. Pathophysiological hallmarks of dystonia, including prolonged tactile temporal discrimination thresholds and reduced brainstem blink reflex inhibition, are found in dystonic tremor but not essential tremor ^17, 18^. There is emerging evidence that dystonic tremor improves less than essential tremor with thalamic deep brain stimulation (DBS) ^19^. Recently, phase-specific thalamic DBS has been shown to be more efficient than conventional DBS for essential tremor, but the positive effects were smaller and inconsistent in dystonic tremor ^20^, suggesting fundamental differences in tremor networks between essential and dystonic tremor. Difficulties in achieving satisfactory control of dystonic upper limb tremor, particularly proximal components, and head tremor with thalamic DBS have led to interest in alternative targets, particularly the caudal zona incerta/posterior subthalamic area ^21, 22^. Most recently, magnetic resonance imaging (MRI)-guided focused ultrasound (MRgFUS) thalamotomy has emerged as a promising less-invasive alternative to DBS for disabling dystonic tremor ^23^.

## The role of the cerebellum in dystonia

In recent years, the traditional view of dystonia as being primarily a disorder of the basal ganglia has been broadened to recognize the role of the cerebellum in dystonia pathophysiology. There are many lines of evidence pointing to cerebellar involvement in dystonia. Dystonia has been recognized to occur in focal cerebellar lesions ^24, 25^ and in the setting of genetic degenerative cerebellar disease ^26– 28^. Early attempts to ameliorate severe acquired dystonia included ablative surgery and chronic stimulation targeting the cerebellum. Cooper *et al*. reported chronic cerebellar cortical stimulation to be beneficial for athetosis (dystonia) and spasticity in cerebral palsy ^29^. Interestingly, the improvement in dystonia, unlike in spasticity, was delayed, analogous to progressive improvement in dystonia seen following pallidal DBS. Functional imaging studies, which have elucidated abnormal network activity in dystonia, have frequently demonstrated the cerebellum as an important node within pathological network activity ^30, 31^. Diffusion tensor imaging studies have demonstrated abnormal integrity of cerebello-thalamic tracts in *DYT1* and *DYT6* ^32^. Eyeblink auditory conditioning, a paradigm mediated by the olivo-cerebellar pathway, is abnormal in focal dystonia ^33^ but not in *DYT1* or *DYT6* dystonia ^34^, suggesting differential cerebellar involvement in different dystonia subtypes. The presence of pallido-cerebellar coupling, inversely correlated with severity of dystonic symptoms, has been demonstrated by using a combination of pallidal local field potentials and magnetoencephalography ^35^. In a murine pharmacological model of rapid-onset dystonia parkinsonism, chemical blockade of sodium pumps with ouabain in the basal ganglia induces parkinsonism without dystonia, whereas injections in the cerebellum induce ataxia and then dystonia, and the ensuing dystonia is preventable by lesioning the cerebellar–basal ganglia connections in the contralateral thalamic nucleus ^36^. In such mice rendered dystonic by cerebellar ouabain injection, striatal recordings disclose high-frequency discharge bursts, resembling those seen in human dystonia, which, along with accompanying dystonia, are abolished if the cerebellar–basal ganglia relay in the contralateral thalamus is lesioned ^37^. In the *DYT1* mouse, cerebellar synaptic maturation is impaired ^38^, and reduction of Torsin A production in healthy mice by injection of viral vectored short hairpin RNAs (shRNAs) produces dystonia for cerebellar but not basal ganglia injection ^39^, providing potential mechanisms for dysfunction in human *DYT1* dystonia.

Further evidence for a cerebellar contribution to dystonia has come from recent studies demonstrating improvement in dystonic symptoms in patients with cervical dystonia following theta-burst transcranial magnetic stimulation (TMS) targeting the cerebellum ^40, 41^. The cerebellum is also being revisited as a therapeutic target for DBS, and there is improvement in dystonia and spasticity in patients with cerebral palsy following DBS of the deep anterior cerebellum ^42^.

## Deep brain stimulation for the treatment of dystonia

DBS of globus pallidus internus (GPi) is well established for the treatment of isolated idiopathic or genetic (primary) generalized, segmental, and focal dystonias ^43– 45^, and studies with longer follow-up have confirmed long-term benefit ^46^. Acquired combined (secondary) dystonia may also benefit from GPi DBS, but the clinical improvement is less and more variable ^47– 49^. This distinction between superior benefit of “primary” versus “secondary” dystonia, though broadly true when dystonias were formerly classified in this way, is less relevant or accurate under the revised dystonia classification ^1^. For example, some acquired combined dystonias such as tardive dystonia improve to a similar degree as isolated idiopathic or genetic dystonia ^49, 50^, and certain genetic combined dystonias such as X-linked parkinsonism dystonia (Lubag/ *DYT3*) ^51, 52^ and *NBIA/PKAN* ( *PANK2*) may respond very well to DBS ^53, 54^. Conversely, some types of idiopathic isolated dystonia, particularly involving the face or larynx, may respond less or more variably ^55^. For these reasons, differing forms of dystonia respond differently to DBS in both the isolated and the combined groups, depending on the etiology and dystonia distribution, and dichotomizing outcomes with respect to older concepts of “primary” and “secondary” may be overly simplistic.

Many patients with acquired and combined dystonia have structural brain lesions visible on MRI, particularly involving the basal ganglia, which may help predict a poorer response to DBS ^47^. Predicting DBS outcome is more difficult in acquired combined dystonia because of the limited and variable benefit and the presence of combined features, including spasticity and cerebellar or sensory deficits, which are usually DBS resistant. A recent study evaluated the utility of somatosensory evoked potentials and central motor conduction time in pediatric dystonia prior to DBS and found abnormalities in these studies to be useful for predicting poorer outcome or treatment failure ^56^. Genetic testing may also provide important information in stratifying DBS outcome ^57^, as some genetic dystonias such as *DYT1* (Torsin A) and *DYT11* ( *SCGE*) are highly DBS responsive ^48, 58– 60^ whereas *DYT6* ( *THAP1*) may show more modest or variable improvement ^61– 63^.

A recognized limitation of GPi DBS is the unwanted side effect of slowness (bradykinesia/akinesia) and other Parkinson-like motor symptoms in a small proportion of patients ^58, 64– 66^. Although bradykinetic side effects of GPi DBS in dystonia may be reversible with adjustment of DBS electrical parameters, it is sometimes difficult to abolish side effects without compromising clinical benefit for dystonia. This limitation of GPi DBS has led to interest in alternative DBS targets in dystonia, and the most promising reported results have been for subthalamic nucleus (STN) stimulation, which has been shown to provide effective relief of isolated cervical, segmental, and generalized dystonia ^67– 69^. Although STN DBS does not induce bradykinesia, dyskinesia is a very frequent side effect but responds to DBS reprogramming ^67^. In acquired dystonia, STN provides a useful alternative in patients in whom the pallidum has been damaged precluding surgical targeting of GPi ^70^. The continued interest in and development of new DBS targets in dystonia are important because of therapeutic limitations of GPi DBS and potential improvements in side effect profiles and energy consumption allowing extended DBS device life span. Beyond targets, recent developments in adaptive DBS for Parkinson’s disease ^71^ herald similar potential in dystonia, provided that a suitable electrophysiological signal in dystonia for DBS modulation can be identified. Recently, low-frequency synchronized oscillations in the theta 3–7 Hz range identified in the basal ganglia thamalo-cortical circuits in dystonia correlating closely with dystonic movements appear to be a promising candidate as a physiological biomarker for adaptive DBS ^72^. The identification of abnormal networks of brain activity in dystonia ^73^ raises the interesting possibility of customized approaches to dystonia DBS in the future where specific network nodes are targeted singly or multiply for neuromodulation, using adaptive stimulation to normalize brain network activity.

## Stereotactic lesions for the treatment of dystonia

Stereotactic lesional interventions, having been almost abandoned after the advent of DBS, are experiencing a renaissance owing to the recent development of MRgFUS as well as renewed interest in stereotactic radiosurgery ^74, 75^. These developments create potential for new and less-invasive stereotactic lesional treatment approaches for dystonia. MRgFUS has been successfully applied in the treatment of essential tremor by unilateral thalamotomy ^76, 77^ and Parkinson’s disease by unilateral thalamotomy ^78^ and subthalamtomy ^79^. The first longer-term studies in essential tremor indicate that benefits remain stable over time ^80^. Currently, there is no evidence to support the use of MRgFUS stereotactic lesions for the treatment of dystonia, and its utility, at this stage, remains speculative.

Targeting the pallidum with MRgFUS, though currently unproven, may be a potential treatment option for dystonia in the future. In the past, bilateral surgical pallidotomy was found to be effective in generalized dystonia ^81, 82^ but was superseded by DBS. Recently, bilateral surgical pallidotomy has been revisited in cranio-cervical dystonia with good effect ^83, 84^. For MRgFUS pallidotomy in dystonia, problems yet to be overcome include perfecting GPi lesions without causing optic tract injury and establishing the safety of bilateral lesions. Moreover, MRgFUS pallidotomy for dystonia, either unilateral or bilateral, remains investigational within clinical trials. For focal and unilateral forms of dystonia, MRgFUS thalamotomy may have a role. Surgical thalamotomy has been used successfully for focal task-specific hand dystonias ^85, 86^, and emulating this procedure with MRgFUS may deliver similar benefits. To date, there are no published studies of MRgFUS in dystonia, but one clinical trial (ClinicalTrials.gov Identifier: NCT02252380) is evaluating MRgFUS for a range of movement disorders, including dystonia.

## Dystonia pathophysiology: insights from neurophysiological studies

In the same way that conceptual shifts have challenged the traditional view of dystonia as a disorder of basal ganglia to include contributions from the cerebellum, the understanding of dystonia pathophysiology has evolved. The traditional view of dystonia as a disorder characterized by multi-level neural disinhibition ^87– 90^ has been refined to incorporate abnormal sensory processing ^91– 93^ and altered motor cortex plasticity ^94^. Although consistent patterns of neurophysiological abnormality exist in dystonia, it remains unclear the extent to which these abnormalities *cause* dystonia, create the conditions in which dystonia can develop (endophenotype), or represent a by-product of the dystonic movements (epiphenomenon). For example, neurophysiological abnormalities frequently can be identified in non-dystonic body parts or their homologous brain regions ^89, 95, 96^, can be present in non-manifesting dystonia gene carriers ^97^, and do not always correlate with dystonia severity or improvements after treatment. In acquired dystonia, some neurophysiological hallmarks of isolated idiopathic or genetic dystonia are lacking ^98^, suggesting that they are not a prerequisite for the development of dystonic movements. The presence of several different types of abnormality also raises questions as to which is the most important or fundamental. One of the most consistent sensory abnormalities in dystonia is impaired sensory temporal discrimination threshold (STDT) ^93, 99, 100^. Impaired STDT occurs with lesions involving the primary somatosensory and parietal cortex, thalamus, and striatum ^101^ and in dystonia has been linked to defective inhibition within the somatosensory cortex ^102, 103^. Impaired STDT not only discriminates between dystonia patients and healthy subjects ^96^ but also is present in non-manifesting *DYT1* gene carriers ^104^ and in asymptomatic first-degree relatives of dystonia patients in a frequency similar to that expected for a dominant gene with incomplete penetrance ^105^. Collectively, these data point to central timing defects (reflected in impaired STDT) playing an important part in dystonia endophenotypes and suggest that STDT provides a potential biomarker for dystonia susceptibility and dystonia gene carriage in unaffected relatives of patients with dystonia ^106^. An additional piece of evidence supporting a permissive or endophenotypic role for impaired STDT in dystonia is that STDT abnormalities are not reversed by effective therapies for dystonic symptoms such as DBS ^107^ or botulinum toxin ^108^. Of recent particular interest, the superior colliculus, a structure implicated in central timing circuits ^109, 110^ and dystonia ^111^, has been shown to exhibit abnormally reduced functional MRI activity correlated with impairments in STDT in patients with cervical dystonia ^112^.

## Dystonia genetics: recent discoveries and highlights

Recently, a revised nomenclature for genetic dystonias has been proposed which replaces the numerical designation of each dystonia ( *DYT1*, *2*, *3*, …n) with a suffix denoting the gene responsible; for example, *DYT11* becomes *DYT-SCGE* ^113^. These revisions offer a number of advantages but have not yet been universally adopted, so the traditional nomenclature is used in this review. Until quite recently, there were only two isolated dystonia genes identified: Torsin A ( *DYT*) discovered in 1997 ^114^ and *THAP1* ( *DYT6*) discovered in 2009 ^115^. This list has expanded with the discovery of *GNAL* ( *DYT25*) ^116^, *ANO3* ( *DYT24*) ^117^, and *CIZ1* ( *DYT23*) ^118^. A major breakthrough has been the discovery of *TUBB4A* as the cause of *DYT4* “whispering dysphonia” ^119^. *TUBB4A* mutations also cause hypomyelination with atrophy of the basal ganglia and cerebellum (H-ABC) ^120^, and intermediate phenotypes with features of both H-ABC and DYT4 have been described ^121^. *TUBB4A* mutations disrupt microtubular function ^122^, and abnormal accumulation of microtubules in oligodendrocytes has been demonstrated in human and rodent models of *TUBB4A* hypomyelination ^123^. The wide phenotypic spectrum may be explained by the discovery that the specific *TUBB4A* mutation determines the extent of neuronal and oligodendrocytic involvement, such that neuronal involvement is present in *DYT4* whereas H-ABC is characterized by combined neuronal and oligodendrocytic involvement ^124^. The first two genes linked to recessively inherited isolated dystonia ( *DYT2*) have been identified as *COL6A3* ^125^ and *HPCA* ^126^. However, the specificity of *COL6A3* mutations as a cause of dystonia has been questioned ^127^, and *HPCA* appears to be extremely rare in isolated dystonia cohorts ^128^.

The genetic understanding of combined dystonias has also expanded in recent years. Myoclonus dystonia ( *DYT11*) is usually caused by *SGCE* mutations ^129, 130^ but has also been described with mutations of *KCTD17* ^131^ and *CACNA1B* ^132^. The paroxysmal dystonias and dyskinesias that have been genetically characterized include paroxysmal dystonic choreoathetosis *MR-1* ( *DYT8*) ^133^, paroxysmal kinesogenic dyskinesia *PRRT2* ( *DYT10*) ^134^, paroxysmal choreoathetosis/spasticity ( *DYT9*), and paroxysmal exercise-induced dyskinesia ( *DYT18*); the last two are allelic disorders and are due to mutations in the *SLC2A1* gene encoding glucose transporter 1 ( *GLUT1*) ^135, 136^.

Dystonia may coexist with spinocerebellar ataxia (SCA), parkinsonism, or hereditary spastic paraplegia (HSP). Dystonia in the setting of SCA is most frequently seen with SCA 2, 3, 1, and 6 and may be associated with longer repeat expansions ^26, 137^ whereas *SCA17* can rarely present with dystonia ^27^. Dystonia is also commonly seen in ataxia telangiectasia ^138^ and ataxia with oculomotor apraxia (AOA) type 2, senataxin gene ^139, 140^, whereas in *AOA1* aprataxin gene, chorea is more common ^141^ but dystonia has been reported ^142^. *POLG* mutations with ataxia, neuropathy, or oculomotor palsy may also cause dystonia ^143^. Dystonia and parkinsonism occurs with gene mutations of *PRKRA* ( *DYT16*) ^144, 145^, *GCH1* ( *DYT5a*) ^146^, *TH* ( *DYT5b*) ^147^, *ATP1A3* ( *DYT12*) ^148^, and *TAF1* ( *DYT3*) ^149^. *GCH1*-related dystonia-parkinsonism may be more common than previously thought ^150^. *ATP1A3* has a wide phenotypic spectrum, including not only rapid-onset dystonia parkinsonism ( *DYT12*) but also alternating hemiplegia of childhood ^151^ and CAPOS (cerebellar ataxia, areflexia, pes cavus, optic atrophy, and sensorineural hearing loss) syndrome ^152^ and familial generalized dystonia without parkinsonism ^153^. Dystonia may also be a prominent or presenting feature in predominantly parkinsonian genes, including Parkin ^154^ and *PINK1* ^155^. Dystonia has also been described in several genetic subtypes of HSP, including *SPG7* ^156– 158^.

Advances in genetic technology with next-generation sequencing allowing whole exome sequencing have made substantial inroads in the field of dystonia genetics ^159^. Whole exome sequencing has been used successfully to identify the causative dystonia gene in almost 40% of a cohort of patients with early onset generalized dystonia ^160^. Focal dystonia cohorts, including spasmodic dysphonia (SD), have also been probed by using improved genetic techniques. SD is a focal laryngeal dystonia in which 10 to 15% of patients report a positive family history of dystonia ^161, 162^. In cohorts of SD, identification of a known dystonia gene is rare, but isolated cases of *GNAL* mutations ^163^ or *THAP1* have been identified ^164^, leaving most of the familial cases unexplained. Early onset laryngeal dystonia with progression to generalized dystonia has been associated with *THAP1* ^165^; however, these patients differ clinically from those with conventional later-onset, sporadic, or familial SD, in whom generalized dystonia does not occur. Although the genes responsible for familial SD remain elusive in most cases, there is no doubt that familial clustering in SD may confer biological specificity, as demonstrated by a recent study which showed differences in brain morphology, determined by MRI, between familial and non-familial SD ^166^.

## Psychogenic dystonia: a reappraisal in light of neurophysiological and imaging data

Psychogenic or functional dystonia is a recognizable subtype of dystonia, which can be diagnosed on the basis of positive clinical criteria ^167, 168^. Additional features pointing to psychogenic dystonia include sudden onset, waxing/waning clinical course, variable and distractible motor phenomenology, fixed postures of distal limbs, and the frequent presence of chronic pain ^169– 171^. In many cases of psychogenic dystonia, an initial triggering event such as minor trauma or illness is identified. Like organic dystonia, psychogenic dystonia can be severely disabling and result in fixed joint deformity, tendon contracture, and dependency ^172^. Although many experts consider psychogenic dystonia to be caused primarily by underlying psychiatric or psychological disturbance (most commonly, conversion disorder), many patients with psychogenic dystonia have no identifiable psychiatric or psychological illness, and a proposed revision of the Fahn-Williams criteria ^167^ removes the need for psychiatric disorder to be identifiable ^173^. Moreover, there is significant pathophysiological overlap between organic and psychogenic dystonia. Shared abnormalities between the two conditions include cortical and spinal disinhibition ^174, 175^, abnormal thalamic firing rates and sensory receptive fields ^176^, and impaired sensory temporal discrimination ^177^. Exceptions are TMS paired associative cortical plasticity, which is increased in organic dystonia but not in psychogenic dystonia ^178^, and the blink reflex recovery cycle, which is normal in psychogenic dystonia and abnormally disinhibited in organic blepharospasm ^179^. Functional imaging studies, comparing patients with psychogenic dystonia with healthy subjects, demonstrate abnormal patterns of brain activation different from those seen in organic dystonia but with some shared similarities ^180^. TMS has been reported to be beneficial in treating functional movement disorders, including psychogenic dystonia ^181^, and the probable mechanism of action is cognitive-behavioral adaptation rather than physiological cortical neuromodulation ^182^.

The term psychogenic dystonia has been criticized as being potentially pejorative and over-emphasizing psychiatric mechanisms, and functional dystonia has been proposed as the preferred term ^183^, as it is more acceptable to patients and highlights an underlying disturbance of brain function rather than structure but leaves etiological mechanisms open for future elucidation. How can the demonstrated neurophysiological and imaging abnormalities be reconciled with a disorder that is widely considered to be psychiatric or psychological in origin? It is possible that the abnormalities can be explained by the presence of enduring patterns of dystonic movements, producing secondary brain adaptation (epiphenomenon). The other possibility is that some endophenotypic abnormalities that characterize organic dystonia patients and their asymptomatic relatives are shared by psychogenic dystonia sufferers and serve as a biological predisposing factor to both conditions and that environmental factors, including both physical and possibly psychological trauma, serve as a trigger.

## Conclusions

Although the dystonias are collectively unified by the presence of involuntary movement patterns recognizable as dystonia, they present distinct challenges owing to their clinical, genetic, and pathophysiological diversity. As the understanding of differences between the dystonias improves, so too does the potential for more efficient and targeted therapy. In this review, topics in dystonia have been selected where there have been new discoveries and conceptual shifts, which will help drive therapeutic advances and benefits for dystonia patients in the future.
